# An Ultra High Gain Converter for Driving HASEL Actuator Used in Soft Mobile Robots

**DOI:** 10.3390/biomimetics8010053

**Published:** 2023-01-26

**Authors:** Tirthasarathi Lodh, Hanh-Phuc Le

**Affiliations:** Electrical and Computer Engineering Department, University of California San Diego, La Jolla, CA 92093, USA

**Keywords:** hybrid converter, extremely large conversion ratio, high voltage breakdown, soft-charging, switched-capacitor, voltage multiplier, dickson

## Abstract

Soft robots have the potential to fundamentally change interactions between robots and the surrounding environment, and between robots and animals, and robots and humans in ways that today’s hard robots are incapable of doing. However, to realize this potential, soft robot actuators require extremely high voltage supplies of more than 4 kV. The electronics that can satisfy this need currently are either too large and bulky or unable to achieve the high power efficiency required for mobile systems. To meet this challenge, this paper conceptualizes, analyzes, designs, and validates a hardware prototype of an ultra-high gain (UHG) converter that can support extremely large conversion ratios up to ∼1000× to provide up to 5 kV output voltage from an input voltage of ∼5–10 V. This converter is demonstrated to be able to drive HASEL (Hydraulically Amplified Self-Healing Electrostatic) actuators, a promising candidate to realize future soft mobile robotic fishes, from an input voltage range of a 1-cell battery pack. The circuit topology employs a unique hybrid combination of a high-gain switched magnetic element (HGSME) and a diode and capacitor-based voltage multiplier rectifier (DCVMR) to enable compact magnetic elements, efficient soft-charging in all flying capacitors, and adjustable output voltage capability with simple duty-cycle modulation. Achieving an efficiency of 78.2% at 15 W output power, while providing 3.85 kV output from 8.5 V input, the proposed UGH converter proves to be a promising candidate for future untethered soft robots.

## 1. Introduction

Human life and productivity have seen revolutionary changes in recent decades, to which robots have contributed fundamentally. The integration of robots in everyday activities has also increased at an unprecedented rate with the next generation easily surpassing the previous generations in capabilities, market penetration, and customer satisfaction. One such example is robotic prosthetics, the market size of which is estimated to double from $790.8 million in 2016 to $1.76 billion by 2025, i.e., CAGR (Compound Annual Growth Rate) of 9.2% [1]. The approximate number of conventional robots, typically made of solid and hard materials, is estimated to be around 1.6 million or more in global operation. Hard robots find wide applications in factories for automobiles and electrical products. However, there remain a lot of tasks that have to be performed by human workers, particularly those requiring complex conformal interactions with animals, or humans. Accomplishing these tasks requires a new generation of robots [2] that are constructed with soft, versatile, lightweight, and compact materials. Hence, a lot of recent efforts, from both academic research and industry, have been poured into realizing soft robots for a wide range of applications, for example, soft robotic hands for soft grip [3,4,5,6,7] and sensing [8,9] or soft robots that mimic animal anatomy and operation [10,11,12,13] to better study their structure and movements in a rough environment or underwater [14,15,16].

### 1.1. HASEL Actuator Construction and Fabrication 

**Figure 1:**
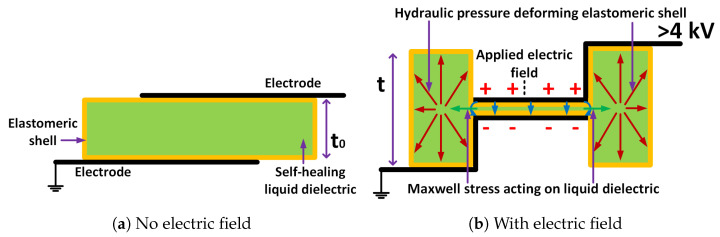
Structure and operation of HASEL [16].

The key component of a soft robot is the soft actuator, one widely used type of which is called Dielectric Elastomer Actuators (DEAs) [17,18]. These actuators can produce high forces, have high power per weight ratios (W/kg), produce large strains (>1000%) [14,15,16], possess high energy density (>3 MJ/m${}^{3}$) [19], exhibit self-sensing [20], and achieve fast actuation rates (10 ms to 1 s) [21]. The HASEL (Hydraulically Amplified Self-Healing Electrostatic) actuator [16], illustrated in Figure 1, is a special type of DEA. There are three basic components listed in Table 1 used to fabricate HASEL actuators.

First, flexible electrodes are patterned on the soft deformable pouch’s exterior. A conductive, and safe carbon-based substance that is used to create ultra-thin layers of elastic electrical conductors is the main component of the electrodes. The electrode material is deposited in exact topologies, utilizing a variety of commercial techniques used in the booming field of flexible electronics. Since a HASEL actuator’s functionality is determined by its electrode geometry, the capacity to design precisely defined electrode patterns is essential for actuator customization.

Second, a flexible, soft, and thin thermoplastic polymer film, which is extensively found in the high-performance capacitor industry, is used to prepare the elastomeric shell. Electric linear actuators made of these materials have the potential to have extremely long cycle lifetimes.

Third, a liquid dielectric makes the self-healing core. Transformer oils are a common type of liquid dielectric, although simple cooking vegetable oils can also be used. To achieve a particular actuator response, the liquid dielectric utilized in HASEL actuators can have a wide range of different characteristics. For instance, the fluid’s viscosity can be adjusted to quicken actuator response times or add passive vibration damping.

It has been shown that one can tailor the performance of these materials to meet the specific needs of different applications. Hence, the materials used in the construction of HASEL actuators are not only low-cost and environmentally friendly, but they are also versatile.

As shown in Figure 1, to operate the HASEL actuator, a voltage is applied between the two electrodes. A large voltage (>4 kV) and, thus, its electric field pull the two parallel electrodes closer together and, thus, push the fluid in between them out to expand the height of the pouches from $t_{o}$ to *t*, as shown in Figure 1b. When multiple actuators are stacked on top of each other, higher strain for the same load is achieved. When a higher voltage is applied to the actuator, a higher load can be lifted.

### 1.2. Stringent Power Supply Requirement for the Soft Robots

While exhibiting many advantageous physical and mechanical characteristics, a key challenge in adopting these soft robots made up of HASEL actuators lies in the electronics to support them, because they require an extremely high electric field to change their shape to create actions [22,23,24,25,26,27]. Since the electromechanical force of dielectric elastomer material is quadratically related to the input voltage, a high supply voltage is required for stronger force and larger payload. Besides this, to enable a fast and frequent response from a soft robot, and to support larger systems with larger payloads, high supply power is also a critical requirement [14,16]. The combination of high supply voltages (≥4 kV), relatively high power (≥5 W), and output modulation poses a critical challenge in designing an efficient, modular, and miniaturized power converter for the actuators, especially when its input voltage is as low as a Li-ion battery voltage of ∼10 V. The input battery compatibility enables next-generation soft robots to be completely untethered [28] and mobile. High efficiency and small size allow soft robots to have longer operation times. A modular design, therefore, is needed to support system scaling up to higher power for heavier payloads and faster operations. These were the key goals of this work.

### 1.3. Literature Review 

There has been a lot of effort in research and industry to design DC–DC converters for extreme conversion ratios and output voltages. They can be broadly classified into four categories: (1) switched-capacitor converters, (2) switched-inductor converters, (3) converters using a transformer, and (4) converters using a coupled inductor. As described in more detail below, each of these categories has its own limitations, such that modifications and synchronous combinations of multiple types of these converters are required to satisfy the required ultra-high gain (UHG) of up to ∼1000× with high efficiency and in a modular compact size.

Switched capacitor converters [29,30] can be compact and can provide a high output voltage. The popular SC converter topologies are series–parallel, Fibonacci, doubler, ladder, hybrid, and Dickson. The key drawback of the SC converter is that its voltage gain depends on the number of components. To achieve a large voltage gain, they require a very large number of components. Hence, switched capacitor converters alone can not be used for ultra-high gain. To achieve a very high gain, the SC converters need to be preceded by another circuitry providing a high gain.

Conventional inductive converters, such as boost, buck–boost, Cuk, sepic, and zeta-derived topologies [31], use only inductors with charging duty cycle modulation to generate voltage conversion. Even though theoretically, it is possible to obtain an extremely high voltage gain from these converters, their practical, efficient voltage gain is only limited to 2–3 times, due to the parasitic components present in the circuits. These types of converters find wide application in mobile applications, regular consumer electronics, and low-voltage DC microgrids with renewable energy sources like photovoltaic (PV) solar cells, fuel cells, and storage sources, like batteries and ultracapacitors. In another effort to increase voltage gain, the converter reported in [32] generated a positive as well as a negative output and took the final output between these two outputs. This approach achieved approximately double the output voltage compared to the previous family of converters, but not enough for an extremely high voltage gain of >1000×. It is quite evident that the duty cycle modulation alone with an inductor is not sufficient for ultra-high voltage gain.

To increase the voltage gain, a different family of converters has been reported on in [32,33,34,35,36,37], which combines a boost or buck–boost type of topology with diode and capacitor-based voltage multiplier rectifiers (DCVMR). Even though the DCVMR stage provides additional voltage gain, its ideal gain increases linearly with the number of levels (equivalent to the number of diodes). That means it still requires many levels and, hence, many components to achieve high voltage gain, leading to design complexity and adverse cost trade-offs. Furthermore, for some of the DCVMR configurations, there is an optimum gain point, beyond which the voltage gain has diminishing returns, even if the number of levels is increased, because of the significant cumulative voltage drop of the flying capacitors.

Another common approach to achieving large voltage gain is to use a transformer, as in forward, push–pull, half-bridge, or full-bridge converters. Reasonable step-up voltage gain can be achieved by increasing the turn ratio of the transformers. However, the duty cycle has to be less than 50 percent to allow transformer core resetting in the forward converter and avoid the shoot-through problem in push–pull, half-bridge, and full-bridge converters. Hence, the duty cycle brings the voltage gain down by a factor of 2 or more. As a result, a very high value of the transformer turn ratio is the only way to achieve a high voltage gain that leads to undesirably large transformer sizes. Inductors can be placed at the converter input to make configurations like current fed push–pull, half-bridge, and full-bridge structures [38]. This arrangement provides a boost-type conversion ratio. Hence, for the same overall voltage gain, the turn ratio requirement can be reduced by 4× or more compared with a simple transformer strategy. However, in order to achieve the required voltage gain of ∼1000×, the transformer needs to support 240× gain, which is also its turn ratio, making it unacceptably large, and also inefficient, in operation.

As an alternative to providing high voltage gains from the inductive side, coupled inductors have been used with high turn ratios for high voltage gain as reported in [39,40,41,42,43,44,45,46,47,48,49,50,51,52,53,54,55,56,57]. These designs tackle the leakage inductance, associated with the coupled inductors, by means of a passive clamp using a diode and capacitor or an active clamp circuitry [47,52]. Although several techniques, including output stacking, are used in this family of converters, their voltage gain is still limited to 10×. Even though the voltage gain can be increased by increasing the coupled inductor turn’s ratio, to achieve a voltage gain of the order ∼1000×, the coupled inductor size needs to be excessively large and it is very inefficient.

To achieve the goal of driving soft robotic HASEL actuators, and to address the drawbacks of the available systems, this work proposes a UHG converter that synchronously combines and optimizes multiple stages and their gains. The rest of the paper is organized in the following manner. Section 2 describes the proposed UHG converter and its operating states. Section 3 talks about the hardware prototype and experimental results, followed by the conclusion of the work in Section 4.

## 2. Proposed UHG Converter and Its Operating States

**Figure 2:**
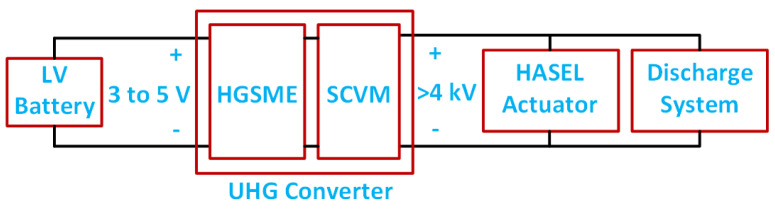
Block diagram of the power system to drive the HASEL actuator.

**Figure 3:**
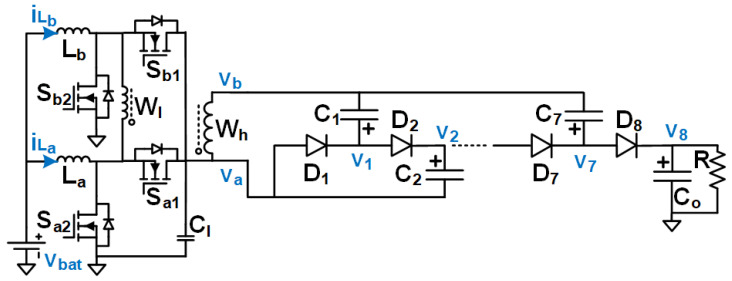
Circuit diagram of the proposed UHG converter.

The block diagram of the power system to drive the HASEL actuator is shown in Figure 2. The proposed ultra-high gain (UHG) converter with two parts is shown in Figure 3. The detailed schematic diagram of the converter is shown in Figure 3. The first part at the input consists of an interleaved boost converter [58] and a transformer that forms the high-gain switched magnetic element (HGSME) part, while a Dickson-based [59] circuit, that forms the diode and capacitor-based voltage multiplier rectifier (DCVMR), is the second part of the converter. In essence, to efficiently provide an extremely large voltage gain, the UHG converter architecture combines multiple smaller voltage gain stages that are synchronously operated. With the DCVMR part providing a significant gain (6×–10×), the HGSME part has a smaller voltage stress and gain requirement, allowing size reduction for magnetic elements.

### 2.1. Operation and Steady-State Analysis of HGSME

**Figure 4:**
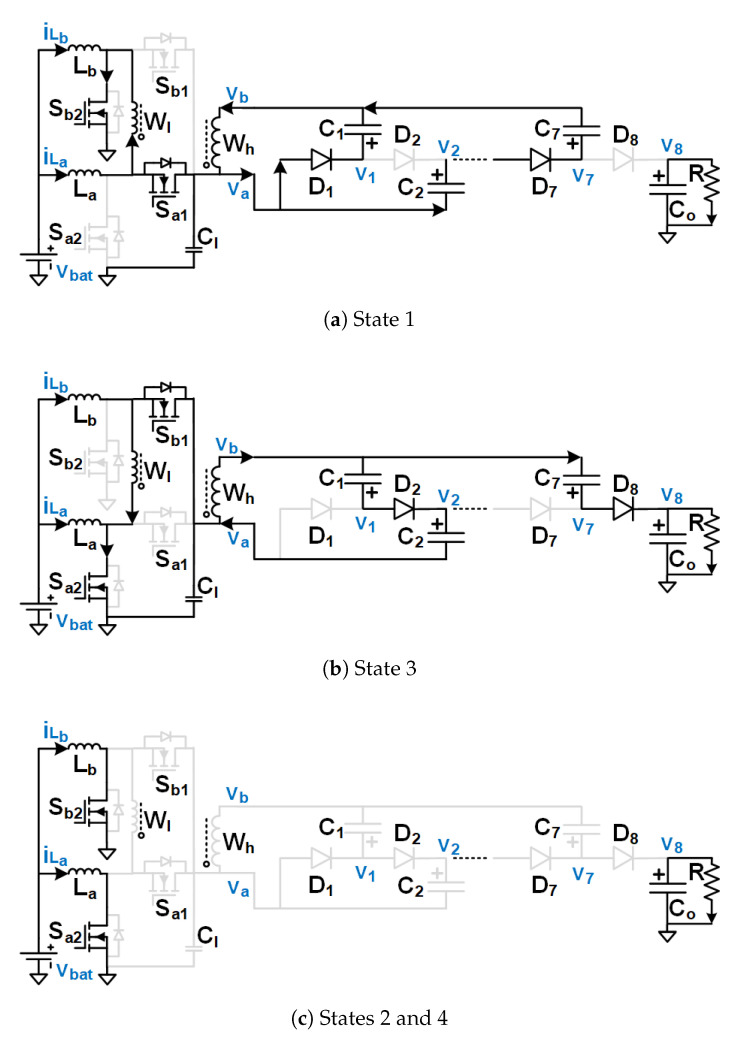
Operating states of the proposed UHG converter.

**Figure 5:**
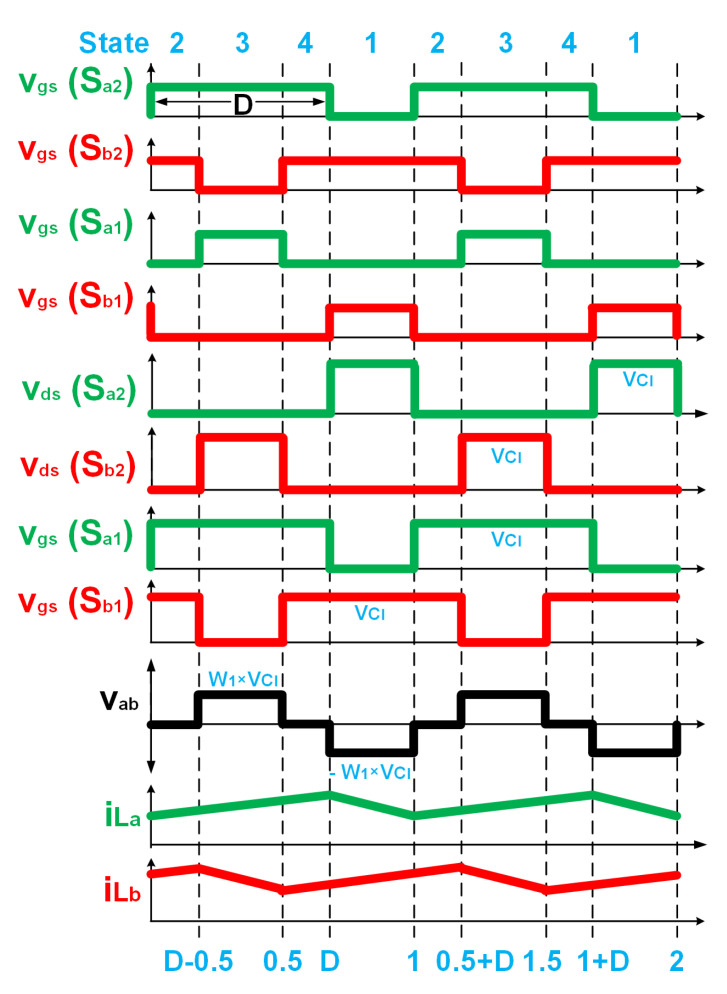
Timing diagram of the proposed UHG converter.

The operating states of the proposed UHG converter, shown in Figure 4, can be explained with the timing diagram presented in Figure 5. The gate to source voltage of switch $S_{a2}$’s ($S_{b2}$) is denoted by $v_{gs}\;\left(S_{a2}\right)$ ($v_{gs}\;\left(S_{b2}\right)$). The drain to source voltage of switch $S_{a2}$’s ($S_{b2}$) is denoted by $v_{ds}\;\left(S_{a2}\right)$ ($v_{ds}\;\left(S_{b2}\right)$). The gate to source voltage of switch $S_{a1}$’s ($S_{b1}$) is denoted by $v_{gs}\;\left(S_{a1}\right)$ ($v_{gs}\;\left(S_{b1}\right)$). The drain to source voltage of switch $S_{a1}$’s ($S_{b1}$) is denoted by $v_{ds}\;\left(S_{a1}\right)$ ($v_{ds}\;\left(S_{b1}\right)$). The two boost converters of the HGSME are operated in two 180-degree-interleaved phases, A and B. Let us assume a duty cycle larger than 0.5 for switches $S_{a2}$ and $S_{b2}$. The switches $S_{a1}$ and $S_{b1}$ operate complementary to the switches $S_{a2}$ and $S_{b2}$ respectively. When the switch $S_{a2}$ ($S_{b2}$) is on, voltage across the switch $S_{a1}$ ($S_{b1}$) is $v_{C_{l}}$, current through the inductor $L_{a}$ ($L_{b}$) rises linearly. When the switch $S_{a1}$ ($S_{b1}$) is on, voltage across the switch $S_{a2}$($S_{b2}$) is $v_{C_{l}}$, current through the inductor $L_{a}$ ($L_{b}$) falls linearly. Hence, the switch voltage is pulse waveforms. The waveforms of the A phase boost converter are 180-degree phase shifted from the corresponding waveforms of the B phase boost converter. The low-voltage primary side of a transformer is connected between the two switching nodes of the two boost converters. Hence, the low-voltage winding (LVW) of the transformer observes the difference between the two switching node voltages $v_{ds}\left(S_{a2}\right)$ and $v_{ds}\left(S_{b2}\right)$, which is a quasi-square wave voltage of peak magnitude $v_{C_{l}}$. The voltage across the secondary winding is a scaled-up version (by a factor of the turn’s ratio of the transformer $W_{1}$) of the quasi-square wave which serves as the input to the DCVMR part.

Using small ripple approximation, assuming continuous–conduction mode (CCM) operation, and applying the Volt-second balance for the inductors $L_{a}$ or $L_{b}$, we can calculate the voltage at capacitor $C_{l}$ in a boost operation as:

(1) $$V_{bat}\times D+\left(V_{bat}-V_{C_{l}}\right)\times \left(1-D\right)=0$$

(2) $$\Rightarrow V_{C_{l}}=\frac{V_{bat}}{1-D}$$

where *D* is the duty cycle of the inductor energizing switches $S_{a2}$ and $S_{b2}$.

The function of the transformer is to amplify the low voltage quasi-square wave at the level of $V_{C_{l}}$ at its LVW by a factor of $W_{1}=\frac{W_{h}}{W_{l}}$ (turn ratio of the transformer). Hence, the peak voltage of the quasi-square wave at the HVW of the transformer is:

(3) $$V_{W_{h}}=\frac{W_{1}V_{bat}}{1-D}$$

The wave shape of the quasi-square wave at the HVW of the transformer $v_{ab}$ can be obtained by amplifying the difference of the waveforms of the drain to the source voltage of the switches $S_{b2}$ and $S_{a2}$:

(4) $$v_{ab}=W_{1}\left\{v_{ds}\left(S_{a2}\right)-v_{ds}\left(S_{b2}\right)\right\}$$

It was observed, from the waveforms of the gate pulses, as seen in Figure 5, that four operating states were in the proposed UHG converter, illustrated in the same Figure. The equivalent circuits depicting the current flow paths corresponding to the different operating states are shown in Figure 4. Equivalent circuits of states 1, 3, and 2 (or 4) are shown in Figure 4a–c, respectively. Those branches of the circuit, which did not carry any current for a particular operating state, were hidden in its equivalent circuit. If the diode was not hidden in a particular branch of an equivalent circuit, it meant that the current flow direction of that branch was from the anode to the cathode side of the diode, because the diode only conducted in the forward-biased condition. During state 1 (3), shown in Figure 4a (Figure 4b), $S_{a2}$ ($S_{b2}$) stayed off, $S_{a1}$ ($S_{b1}$) stayed on, $S_{b2}$ ($S_{a2}$) stayed on, $S_{b1}$ ($S_{a1}$) stayed on, inductor $L_{a}$ ($L_{b}$) was discharged, while $L_{b}$ ($L_{a}$) charged, the current entered through the dotted (non-dotted) terminal of LVW, current left through the dotted (non-dotted) terminal of HVW. In other words, the current left HGSME through the node $v_{a}$ ($v_{b}$) and entered HGSME through the node $v_{b}$ ($v_{a}$). During the identical States 2 and 4 in between States 1 and 3, both switches $S_{a2}$ and $S_{b2}$ stayed on, both $L_{a}$ and $L_{b}$ became charged, zero voltage, as well as zero current, were observed both at the LVW and HVW of the transformer.

### 2.2. Operation and Steady-State Analysis of DCVMR

The high voltage quasi-square wave of peak magnitude $W_{1}v_{C_{l}}$ at the high-voltage winding (HVW) of the transformer was rectified and stepped up simultaneously through the 8PDDCVMR. The 8PDDCVMR consisted of 7 flying capacitors ($C_{1},C_{2},\ldots ,C_{7}$) and 8 diodes ($D_{1},D_{2},\ldots ,D_{8}$). $C_{o}$ was the output capacitor. A linear voltage multiplication from each stage in the 8-level positive output Dickson DCVMR (8PDDCVMR) [60,61]. During State 1 (3), the current entered 8PDDCVMR through the node $v_{a}$ ($v_{b}$) flowed through the odd (even) indexed diodes, charged the even (odd) indexed capacitors, discharged the odd (even) indexed capacitors of the 8PDDCVMR, and left 8PDDCVMR through the node $v_{b}$ ($v_{a}$). During the identical States 2 and 4 in between States 1 and 3, all diodes of the 8PDDCVMR, $D_{1}$-$D_{8}$, stayed off, all flying capacitors of the 8PDDCVMR, $C_{1}$-$C_{7}$, stayed idle. The output capacitor $C_{o}$ discharged and supplied the load current.

With a small ripple approximation, we could get the average capacitor voltages. Applying KVL to all the mesh/loops starting at the node $v_{a}$ and ending at the node $v_{b}$, we obtained the following expressions (∀n≤4):

When current enters through node $v_{a}$ of DCVMR Figure 4a,

(5) $$V_{C_{1}}=V_{W_{h}}$$

(6) $$V_{C_{2n-1}}-V_{C_{2n-2}}=V_{W_{h}}$$

When current enters through node $v_{b}$ of DCVMR Figure 4b,

(7) $$V_{C_{2n-2}}-V_{C_{2n-4}}=V_{W_{h}}$$

It can be observed that the difference between two consecutive capacitor voltages was constant. Hence, they formed an arithmetic progression with the first term as well as the common difference of $V_{W_{h}}$. Hence, the average voltage of the $j^{th}$ (∀j≤7) capacitor was:

(8) $$V_{C_{j}}=jV_{W_{h}}=jW_{1}\frac{V_{bat}}{1-D}$$

The voltage difference between the node $N_{j}$, which was the common node between the diode $D_{j}$ and capacitor $C_{j}$, and the ground was denoted by $v_{suffix}$. Hence, (∀n≤4), the waveforms of the node voltages of the 8PDDCVMR to the ground could be obtained by the following addition:

(9) $$v_{2n-1}=v_{C_{l}}+v_{ab}+v_{C_{2n-1}}$$

(10) $$v_{2n}=v_{C_{l}}+v_{C_{2n}}$$

As the result from Equations (2), (8) and (10), the average voltage of the output capacitor $C_{o}$ was:

(11) $$V_{C_{o}}=\frac{V_{bat}}{1-D}+8W_{1}\frac{V_{bat}}{1-D}=\left(1+8W_{1}\right)\frac{V_{bat}}{1-D}$$

Hence, the ideal voltage gain of the converter in CCM was $\frac{1+8W_{1}}{1-D}$. It can be observed that the voltage gain of the proposed UHG converter was the multiplication of multiple voltage gain stages in a series, including the boost stage, transformer stage, and the DCVMR stage stacked on top of the boost output. As a result, the transformer only needed to contribute a small fraction of the total voltage gain and needed to handle only a fraction of the total output voltage, which allowed a significant reduction of the transformer size.

## 3. Hardware Prototype and Experimental Results

### 3.1. Design of Hardware Prototype

**Figure 6:**
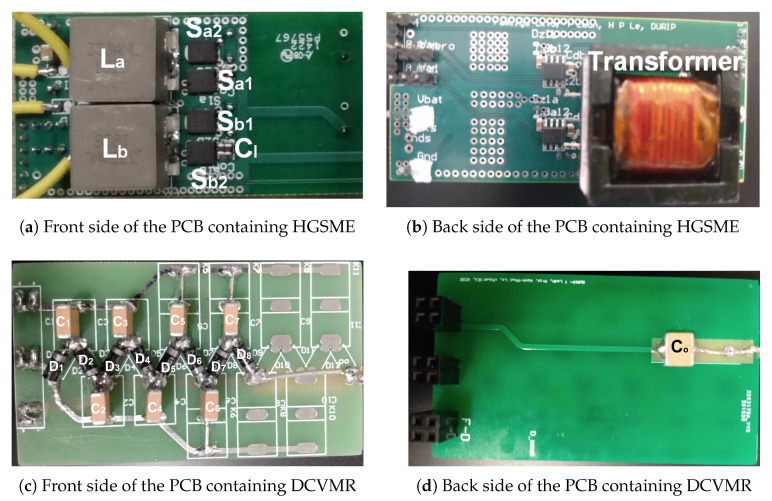
The UHG converter hardware prototype.

A prototype of the proposed UHG converter was implemented on a printed circuit board (PCB), shown in Figure 6, using the components listed in Table 2. In this demonstration, the 8PDDCVMR used 7 flying capacitors forming an ideal voltage gain of 8× with 8PDDCVMR. Since they were on the low-voltage side and relatively easy to implement, nMOS transistors, denoted $S_{a1}$ and $S_{b1}$, were the high-side switches. Half-bridge gate driver ICs were used to drive the switch pair $S_{a2}$ and $S_{a1}$ ($S_{b2}$ and $S_{b1}$).

Challenges to the practical implementation of the UHG converter are the following: the selection of small-size components, both active and passive, to satisfy extremely high voltage blocking without degradation of electrical parameters. For example, it is hard to find capacitors in small package sizes, such as surface-mount devices (SMDs), that can support extremely high voltage ratings and, at the same time, hold high capacitance values. To make it even more challenging, their capacitance value drops drastically with an increase in the applied voltage across them, leading to a decrease in power transfer and efficiency. On the other hand, the footprint of the input inductors becomes large if they need to support a large peak current and, at the same time, have a small ESR. A high magnetizing inductance is desired for the transformer, to keep the current ripple limited to a low value and to facilitate using inductors with a low peak current rating for the same average current. Low current ripple helps in keeping the conduction loss low. The duty cycle of the converter should not be too high (≥0.85). Otherwise, the efficiency of the interleaved boost-based HGSME part drops drastically because of excessive conduction loss of the input inductors. To meet these constraints in the HGSME part, while still providing a high overall voltage gain, the transformer still needs to support a relatively high voltage gain of 30×. It is challenging to find a compact transformer that has such a high turns ratio (hence, a small number of turns on the low voltage side) while having a high magnetizing inductance. Therefore, a custom transformer was designed, optimized, and manufactured, employing $W_{1}=$ 1:30 turns-ratio and EE core that factored in these trade-offs and specifications.

### 3.2. Compact PCB Design for Extremely High Voltage

A key part of this design was to support the extremely high output voltage while minimizing the total PCB area and implementation volume. For this goal, it was very important to eliminate any possibility of any high voltage breakdown and electric arcs that could damage the system. To do this, one needs to do the following: (1) reduce the electric field intensity, and (2) strengthen the dielectric medium between high-voltage nodes in the circuits. Several related methods and best practices to enhance long-term reliability, important for the design, are highlighted below.

#### 3.2.1. Reduction of Electric Field Intensity

##### Geometry of PCB Pads and Solder Joints

The shape of the pads, instead of being rectangular, was rounded off with the highest possible curvature. This resulted in a more uniform distribution of the electrical field lines [62,63], and significantly reduced the localized electrical field intensity, and associated electrical breakdown probability. Joint soldering was done wherever possible with the highest possible radius to reduce the arcing and power loss due to corona [64,65,66]. In this design, a symmetric structure was employed for the component placements from the outputs of the coupled inductors, $V_{a}$, and $V_{b}$, to the output $V_{o}$ such that there was a gradual voltage rise from one node of the board to other adjacent nodes and no vicinity between any high and low voltage nodes.

##### Physical Distance between Nodes

Enough distance had to be kept between different voltage nodes on the high-voltage part. The distance between two nodes can be calculated in two ways: (1) through the medium of air, which is termed clearance, and (2) along the surface of the PCB material, which is termed creepage [67,68,69,70]. Due to the accumulation of dirt, dust, absorption of moisture, solder flux residue, and numerous other reasons, there was a much higher probability of high voltage breakdown along the PCB surface, compared with air clearance. Hence, the recommended creepage distance was at least 2–3 times the clearance. The boards, which were designed with high clearance and creepage distance, could operate at much higher voltage without any electrical arcing with the cost of large board size. On the other hand, arcing was observed at a lesser voltage in the boards which were made more compact. The design shown in this paper experienced several iterations to closely follow these design methodologies to avoid arcing in this extremely high-voltage design while maintaining a small design size.

#### 3.2.2. Strengthening the Dielectric Medium

To make the design compact, there were large voltage differences between the nodes in the same layer (intra-layer) as well as different layers (inter-layer). Hence, both inter-layer and intra-layer dielectric breakdowns required serious consideration.

##### Inter-Layer Dielectric

PCB material, FR4, which has a dielectric strength of 20 MV/m, is very popular and inexpensive. Hence, DCIH converter prototypes have been implemented using FR4. However, it can suffer from degradation of dielectric strength with aging, weak sidewall structure, low soldering temperature, and incapability of restoring the dielectric strength after arcing. For a more compact design and reliable long-term performance, a better, but more expensive option is high-voltage polyimide film (HVPF) which has a much higher breakdown strength of 3000 V/mil and a much stronger sidewall structure compared to conventional FR4. Moreover, PCB thickness can be significantly reduced with HVPF [71]. Between these two options, one can choose other materials, such as Bismaleimide–Triazine resin (BT–Epoxy), ISOLA POLYIMIDE, etc. to achieve a compromise between performance and cost.

##### Intra-Layer Dielectric

A solder mask is used for protecting copper track and isolation in low voltage implementation. However, there is also a probability of it trapping air bubbles or other forms of impurities that could cause surface leakage and partial discharge to accelerate the electrical breakdown process, impairing the board insulation capability between high-voltage nodes. In this design, the solder mask was removed in high-voltage areas of the board and high-voltage nodes were kept in a single layer to avoid this problem. To further improve insulation performance, one could use multiple coatings of Acrylic resin [71]. Additional measures, such as using gold instead of copper to reduce metal degassing, conformal coating, and potting [72], could also be used to further improve the board’s performance and safety [73].

### 3.3. Discussion on Experimental Results

**Figure 7:**
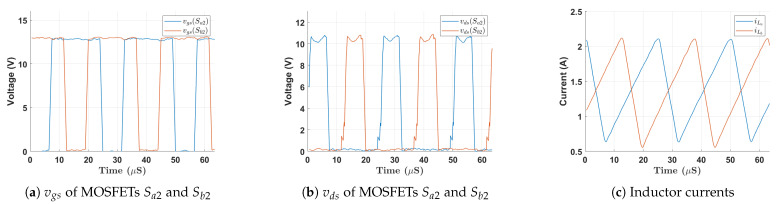
Experimental waveforms during steady state operation of HGSME.

The UHG converter hardware prototype on a printed circuit board (PCB) is shown in Figure 6, including Figure 6a,b showing the front and back side of HGSME, while Figure 6c,d shows the front and back side of 8PDDCVMR implementation. Figure 7 shows the experimental waveforms of the proper functioning of the HGSME part of the proposed UHG converter at the steady state. It can be observed that the waveforms of the two boost converters were 180-degree phase-shifted, as expected.

**Figure 8:**
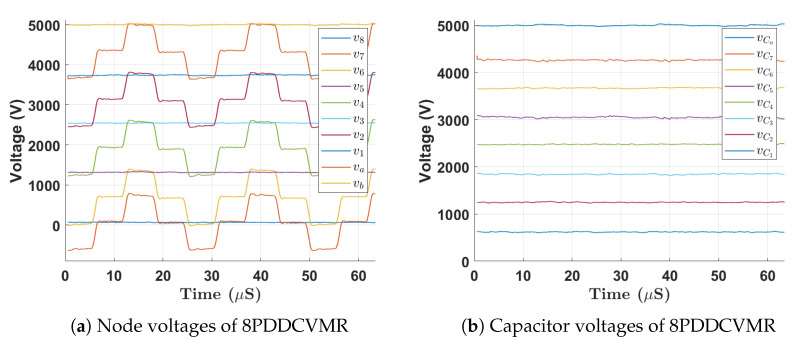
Experimental waveforms during steady state operation of 8PDDCVMR.

Figure 8 shows the experimental waveforms of the proper functioning of the 8PDDCVMR part of the proposed UHG converter at the steady state generating an output voltage of 5 kV. In Figure 8 the even and odd node voltages of the 8PDDCVMR corresponded to Equations (9) and (10), respectively. Voltage swings at the odd switching nodes were observed to be the peak of the quasi-square voltage at the HVW of the transformer which was given in Equation (3). Additionally, in Figure 8b, the flying capacitor voltages were at the right voltage levels, as predicted in Equation (8). $v_{o}$ in Figure 7 shows an output voltage of 5 kV from 5.6 V input, which was correct, according to Equation (11).

**Figure 9:**
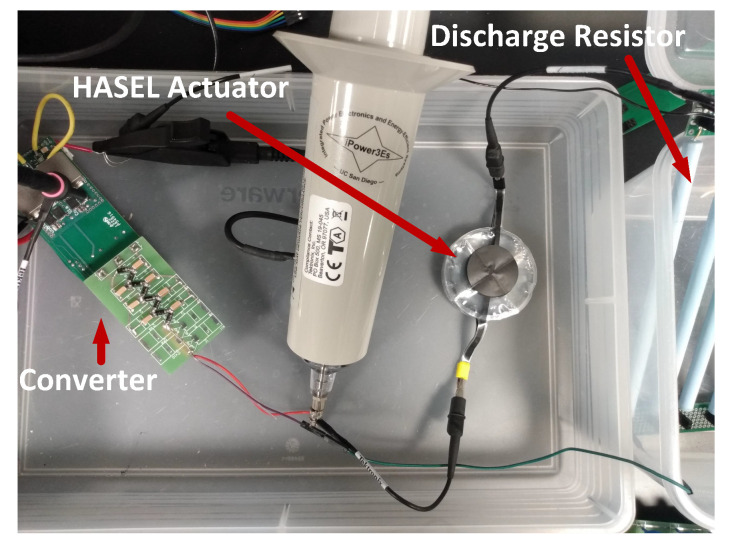
Experimental setup of UHG converter with HASEL with HASEL actuator [16].

**Figure 10:**
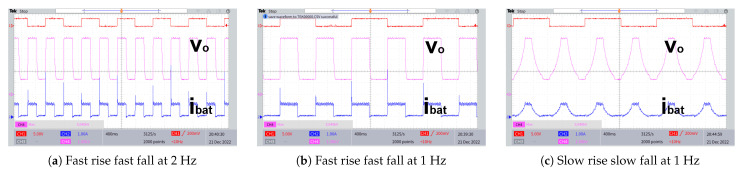
Experimental waveforms of the actuation of capacitive load by the proposed UHG converter.

**Figure 11:**
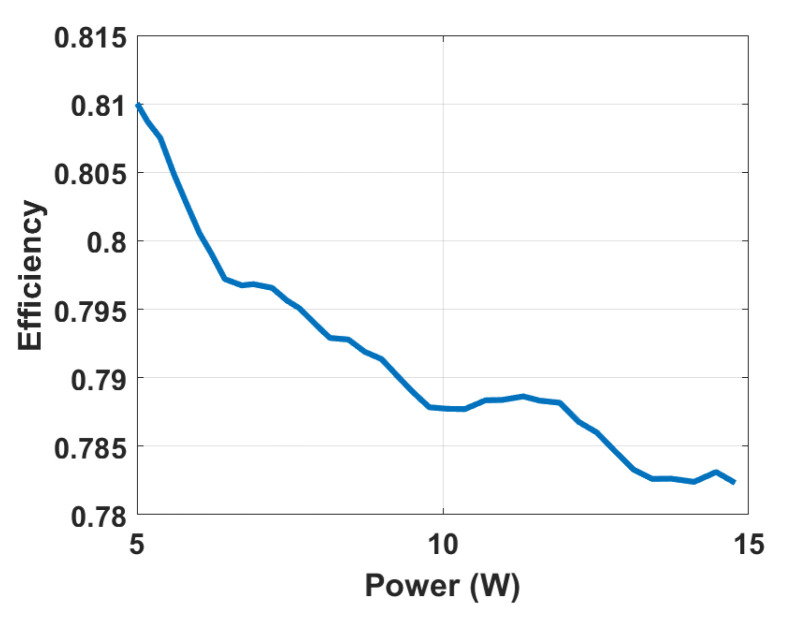
Power vs efficiency of the proposed UHG converter.

Figure 9 shows the Experimental setup of the UHG converter with the HASEL actuator and discharge resistance. Figure 10 demonstrates the experimental waveforms of the proposed UHG converter prototype operating well when actuating (periodically energizing and de-energizing) a capacitive load equivalent to the HASEL actuators at mechanical frequencies of 1 Hz and 2 Hz. These experimental waveforms demonstrated the proposed UHG converter’s correct operation. Figure 11 shows the Power vs efficiency of the proposed UHG converter at duty cycle 0.5 under the open loop. Keeping the load resistance fixed, the input voltage is ramped to generate different points in the plot. Table 3 shows the comparison of the proposed UHG converter with similar commercial products.

## 4. Conclusions

In this paper, a novel UHG is presented with its topology, operation, and experimental results. Response: Thanks to the efficient synchronous operation between two boost converter-based circuits and a Dickson star-based DCVMR circuit, the proposed converter achieved multiple challenging requirements of extremely large conversion ratio, size constraint, efficiency, and regulation simultaneously, which validated its superiority over the available converter products for soft robotic applications. A converter prototype board was implemented, achieving 5 kV from a ∼10 V input.

## Figures and Tables

**Table 1:** Materials used in HASEL Actuators.

Location	Material
Electrodes	Conductive, and safe carbon-based substance
Pouch	Flexible, soft, and thin thermoplastic polymer film
Liquid dielectric	Transformer oil, vegetable oil

**Table 2:** Components.

Part Name	Part Number	Rating	Unit Weight (g)
Half-bridge gate driver	UCC27201DR	120 V, 3 A	0.7
Low-side gate driver	IX4427NTR	34 V, 1.5 A	0.54
Decoupling capacitor	C0805C105K3RAC7210	25 V, 1 uF	0.006
Bootstrap capacitor	C0805C104K3RACTU	25 V, 0.1 uF	0.006
$C_{l}$	C2012X5R1V226M125AC	35 V, 22 uF	0.006
$S_{a1},S_{a2},S_{b1},S_{b2}$	SIRA20DP-T1-RE3	25 V, 63 A	0.51
$L_{a},L_{b}$	PA4344.333ANLT	33 uH	10
$W_{1}$	43119 (Pacific Tx. Corp.)	Ratio 1:30	25
$D_{1},D_{2},\ldots ,D_{8}$	GP02-40-E3/73	5 kV, 0.25 A	0.34
$C_{1},C_{2},\ldots ,C_{8}$	HV2225Y332nXMATHV	3.3 nF, 5 kV	1.26

**Table 3:** Comparison with Commercially Available Products.

Features	[74]	[75]	[76]	[77]	[78]	This *
Power (W)	30	1.25	5	0.5	1	15
Weight (g)	520	9.5	45	4.25	8.49	≤100
Power Density (W/g)	0.06	0.13	0.11	0.12	0.12	≥0.15
Output Regulation/Adjustability	Yes	No	No	No	No	Yes

* Commercial packaging of the proposed UHG converter is not done yet. Hence, the sum of all component weights and volumes is listed for the proposed UHG converter.

## Data Availability

Not applicable.

## Abbreviations

The following abbreviations are used in this manuscript:

LV	Low Voltage
HV	High Voltage
HVW	High Voltage Winding
LVW	Low Voltage Winding
HGSME	High-Gain Switched Magnetic Element
UHG	Ultra-High Gain
DCVMR	Diode and Capacitor-based Voltage Multiplier Rectifier
8PDDCVMR	8 Level Positive output Dickson type DCVMR
HASEL	Hydraulically Amplified Self-healing Electrostatic
